# Advocates for African American Elders: Engaging Older Adults in Education and Research

**DOI:** 10.1093/geroni/igaa057.3097

**Published:** 2020-12-16

**Authors:** Karen Lincoln

**Affiliations:** University of Southern California, Los Angeles, California, United States

## Abstract

Advocates for African American Elders (AAAE) is an outreach and engagement program at the University of Southern California, providing culturally competent health education for older African Americans throughout Los Angeles County (LAC). Founded in 2012 to address racial disparities in health outcomes, AAAE partners with community-based agencies, government, and health plans to address the persistent growing needs of older African Americans. AAAE educates and disseminates information about healthcare policies and resources through fact sheets, educational forums, and the AAAE website. It collaborates with local healthcare providers to improve outreach, education, and care, and assesses service needs and resources via surveys in LAC. The program engages in community-based participatory research to provide real-world solutions for improving health outcomes and build community research capacity. This presentation will highlight AAAE’s community-based approach to raising awareness, increasing knowledge and access to healthcare resources, and improving health outcomes for older African Americans and their families.

